# Application of Solid-State Fermentation for the Improving of Extruded Corn Dry-Milling By-Products and Their Protein Functional Properties

**DOI:** 10.3390/life12111909

**Published:** 2022-11-16

**Authors:** Daiva Zadeike, Zydrune Gaizauskaite, Mantas Svazas, Romas Gruzauskas, Valentas Gruzauskas, Jonas Damasius, Grazina Juodeikiene

**Affiliations:** 1 Department of Food Science and Technology, Faculty of Chemical Technology, Kaunas University of Technology, 50254 Kaunas, Lithuania; 2 Food Institute, Kaunas University of Technology, 50254 Kaunas, Lithuania; 3 Institute of Computer Science, Vilnius University, 08303 Vilnius, Lithuania

**Keywords:** corn-milling by-products, protein fractions, extrusion, solid state fermentation, protein modification, functional properties, digestibility, radical scavenging activity

## Abstract

In this study, the effect of solid-state fermentation (SSF) with *Lactobacillus sakei* MI401 and *Pediococcus acidilactici* PA-2 strains on functional properties of extruded (130 °C; 25 rpm) corn-milling by-products (CMB) and their albumin, globulin, and prolamin fractions was evaluated in order to produce stabilized and functionalized food/feed stock. Extrusion resulted in a considerable reduction of microbial contamination of CMB by five log cycles, increased damaged starch, water-absorption capacity, and lowered protein and fat contents by 12.4% and 37%, respectively. The application of SSF for the extruded CMB have been shown to improve the water absorption, foaming, and emulsifying capacity of albumins and globulins and also increased the digestibility and free radical scavenging activity of prolamins. The essential amino acid content (EAA) in CMB and antioxidant activity of prolamins was lowered after extrusion but significantly increased after SSF. The combination of the abovementioned treatments can be confirmed as a prospective functionalization of CMB, capable of potentially enhancing its safety and improving nutritional, biochemical, and technological properties of proteins.

## 1. Introduction

With the increasing demand for a sustainable environment and healthy food, there is a rapidly growing interest in the industry to provide innovative and sustainable solutions, ensuring the safety and nutritional quality of cereal-based raw materials and food products. In this case, the agroindustry by-products can be valorized into functional components by various technological means and biotechnological methods, providing economic and environmental advantages for the development of new food products and feedstocks.

Corn (*Zea mays* L.), being the third primary cereal crop in the world, contains valuable proteins, fats, and dietary fiber. The application of corn-processing by-products in the food or feed industry can decrease the product’s cost [1]. Corn and its milling by-products are mainly used as a lower-nutritional-value animal feedstock even though corn contains high amounts of phospholipids, carbohydrates, proteins rich in carotenoids, and a fiber fraction rich in phytosterols that can be used to improve the nutrition value of food and feed products [2,3,4]. Recently, corn proteins have been identified as a source of peptides indicating specific bioactivity that can be released during hydrolysis induced by proteolytic enzymes or microbial fermentation [5]. These peptides can be used as bioactive components in food/feed formulations because of their health benefits [6].

Consequently, to reduce microbial contamination and to extend the shelf life of corn milling by-products (CMB), it is important to stabilize raw material by applying thermal technologies, ensuring the safety of end-products [7]. Extrusion is a major technology among other processing techniques used for the production of texture-stable and microbiologically safe corn-based food/feed products. However, when submitted to high-temperature treatment, the feeding value of corn grain may change significantly. Thus, it is necessary to assess the effect of thermal treatment on the functional and technological properties of CMB nutritional components. For these reasons, an appropriate technological approach for the valorization and functionalization of CMB would make possible the improvement of its safety characteristics and functional properties.

One of the ways to increase the nutritional value of cereal-based raw material and to improve its functional properties is lactic acid fermentation. Fermentation is one of the most economical methods of producing and preserving foods and is easily employed for cereal processing. Based on the literature, fermentation with lactic acid bacteria (LAB) can modify starch and protein digestibility in cereal-based products and increase their nutrient availability [8,9]. Most of the research so far has focused on the corn prolamin hydrolysis by microbial enzyme, showing its potential to improve protein functional properties, such as solubility, foaming, and emulsifying capacity, allowing such protein products to apply as functional ingredients with an increased antioxidant effect in both food and non-food applications [10]. Evaluating the impact of the extrusion process on nutritional, functional, and antioxidant properties is mainly focused on corn extrudates [11] or studies on the microstructure, bioavailability, and other functional properties of zein proteins [12].

In this study, the effect of extrusion on the chemical composition and functionality of corn-milling by-products (CMB) was analyzed with emphasis on the amino acid profile, albumin, globulin, and prolamin functional properties. Moreover, the impact of solid-state fermentation (SSF) with *Lactobacillus sakei* and *Pediococcus acidilactici* strains on the hydration properties and the foaming and emulsifying capacity of untreated and extruded CMB albumins and globulins as well as digestibility and bioactivity of prolamins were analyzed.

## 2. Materials and Methods

### 2.1. Raw Material

Corn-grits-milling by-products (CMB), mainly consisting of bran and endosperm particles (moisture 11.21%, protein 11.52%, carbohydrates 70.65%, crude fiber 2.72%, fat 2.63%, ash 1.27%) were obtained from the local mill company (Pasvalys, Lithuania). The batch of corn material (50 kg) was stabilized using extrusion cooking at an industrial scale with a one-screw extruder (Parallal Twin Screw Extruder DKM-EII75x28A, Guangdong, China): temperature in the three extrusion zones was 70/90/130 °C, moisture content of raw material was 16%, and feeding rate was 8.2 kg/h. After extrusion, the material was dried at 80 °C to aprox. 12% moisture.

### 2.2. Microorganisms

The lactic acid bacteria (LAB) strains of *Lactobacillus sakei* (MI401) and *Pediococcus acidilactici* (PA-2), previously isolated from spontaneous rye sourdoughs [13], were used for the fermentation of untreated and extruded CMB material. Before the experiment, all LAB strains were multiplied in a MRS broth (Man-Rogosa-Sharpe, CM 0359, Oxoid Ltd., Hampshire, UK) for 48 h at 30 °C temperature.

### 2.3. Experimental Design

The effect of extrusion on the nutritional quality and functionality of corn-milling by-products (CMB) was analyzed. The untreated and extruded CMB material was analyzed for chemical composition and functional properties, such as water absorption, damaged starch, and starch gelatinization degree, and also was used for the isolation of albumin, globulin, and prolamin fractions. Further, the effect of solid-state fermentation (SSF) on untreated and extruded CMB amino acid profile, protein fraction yields, and functional properties of albumins and globulins, such as hydration ability, foaming, and emulsifying capacity, as well as digestibility and bioactivity of prolamins was evaluated.

For the fermentation of CMB at solid0state conditions (SSF), the untreated (UN) and extruded (E) corn material (50 g) and 60 mL of sterile water were mixed with the pure suspension of each LAB strain (2%, *w/w*), containing on average 9.1 log_10_ CFU/mL. The samples were incubated for 48 h at 30 °C under anaerobic conditions. Finally, different batches of fermented (F) CMB samples were prepared: UN_Ls_, UN_Pa_ and E_Ls_, and E_Pa_—untreated-fermented and extruded-fermented with *L. sakei* (Ls) and *P. acidilactici* (Pa), respectively. The pH of samples was measured directly using a pH electrode (PP-15; Sartorius, Goettingen, Germany). For the analysis of the impact of SSF on amino acid profile, the 48 h SSF processing with *L. sakei* strain was applied. Each fermentation procedure was performed twice, followed by analysis of three sub-samples. Fermented samples of untreated and extruded CMB were analyzed for amino acid profile, protein fraction yields, albumin and globulin, water absorption and solubility, foaming and emulsifying capacity, as well as digestibility and bioactivity of prolamins.

### 2.4. Microbiological Analysis

The total number of aerobic microorganisms in CMB samples was evaluated under standard serial dilution method on plate count agar (PCA) (CM0325, Oxoid, Ltd., Hampshire, UK) and expressed as a log_10_ of colony-forming units (CFU) per gram of material [14]. Each sample (10 g) was homogenized with the 90 mL of NaCl (9 g/L solution). Serial dilutions of 10^−4^–10^−8^ were used for the preparation of final sample. The sample solution was spread on the surface of agar in Petri plates that were incubated at 30 °C for 72 h under anaerobic conditions. For the cell number calculation, the plates with more than >300 CFU were reported as unsatisfactory as well as those of less than 30 CFU, with best results in the range of 50–250 CFU/plate. The results were expressed as the mean of three determinations. The limit of detection (LOD) is 1 CFU, and LOQ is 25 CFU.

### 2.5. Determination of Amino Acids Profile

Amino acids were determined by ultrafast liquid chromatography (UFLC) with automated o-phthalaldehyde (OPA)/9-fluorenylmethyl chloroformate (FMOC). The sample preparation and the UFLC analysis was performed according to Jukonyte et al. [14]. The amino acid standards (A9781 Sigma-Aldrich, Darmstadt, Germany) of 0.5 μmol/mL concentration except for L-cystine at 0.25 μmol/mL in 0.2 M sodium citrate, pH 2.2 were analyzed. A five-level calibration set was used, covering a concentration range of 0.006–0.20 μmol/mL except for alanine and cysteine, each covering a concentration range of 0.06–1.00 μmol/mL. All test samples were analyzed twice.

### 2.6. Chemical Analyzes

Raw material was tested for protein, fiber, fat, and ash contents according to the AOAC Official Methods [15]. The crude protein content was determined by Kjeldahl nitrogen (method 920.152), and the percentage of protein was estimated by multiplying the total nitrogen content by a factor of 5.7. Crude fiber content was determined according to the method 978.10. Ash was determined by combustion of the sample in a muffle oven at 550 °C for 24 h (method 942.05). The fat content was determined by 3 h Soxhlet extraction with hexane (method 996.01).

### 2.7. Determination of Xylanase and Protease Activities

For the xylanase and protease activity determination, the CMB sample (5 g) was mixed with 20 mL of 0.1 M acetate buffer (pH 4.5) or 50 mM potassium phosphate buffer (pH 7.5), respectively, and centrifuged (4500× *g*, 20 min). The obtained supernatants were used for the activity assays. Endoxylanase activity was determined spectrophotometrically by the reducing sugar assay [16] using birchwood xylan (0.5%) as substrate. One unit of xylanase activity was defined as the amount of enzyme required to release 1 μmole of xylose equivalents per minute from the birchwood xylan under the assay conditions (37 °C, pH 4.5). The mode of action of corn protease was determined by the Sigma-Aldrich non-specific protease assay using casein (0.65%) as substrate. One unit of protease activity was defined as the amount of enzyme that liberates the equivalent of 1 μg of tyrosine per minute from the substrate under the conditions of the assay (37 °C, pH 7.5). Enzyme activity was reported in terms of enzyme activity units per 100 g of corn material.

### 2.8. Protein Fractionation

Osborn fractionation of CMB proteins was performed according to Malumba et al. with some modifications [17]. For albumin extraction, a defatted CMB sample (10 g) was extracted with distilled water at a ratio of 1:10 (*w*/*v*) by intensive mixing for 1 h. After, the sample was centrifuged for 20 min (8000× *g*, 4 °C) to obtain albumin extract. For globulin extraction, the residue was extracted with 0.5 M NaCl at a 1:10 (*w*/*v*) ratio following the procedure described above. For the prolamin extraction, the residue was mixed with 70% ethanol, stirred at 60 °C for 90 min, and centrifuged. Albumins, globulins, and prolamins were precipitated with 1 M HCl by adjusting the pH to their isoelectric points of 4.1, 4.3, and 4.8, respectively, and kept at 4 °C overnight. The precipitates were centrifuged at 5500× *g* for 15 min and washed twice with distilled water by centrifugation. Protein sediments were neutralized to pH 7 with 0.1 M NaOH and lyophilized at −40 °C for 48 h (condenser temperature −85 °C, pressure 2 × 10^−6^ mPa; Zirbus Technology, Bad Grund/Harz, Germany). The protein powders were stored in a freezer at −18 °C until analysis.

### 2.9. Determination of Albumin and Globulin Functional Properties

The water-absorption capacity and solubility, emulsifying, and foaming properties of albumins and globulins were analyzed according to Silva-Sanchez [18] with some modifications. All analyzes were carried out in triplicate.

#### 2.9.1. Water-Absorption Capacity and Solubility

For water-absorption capacity (WAC) and water-solubility (WS) determination, a 0.5 g (W_0_) of sample in a graduated centrifuge tube was thoroughly mixed with 5 mL of distilled water, and the pH of the suspension was adjusted to 4, 7, or 9 with 0.1 M HCl or 0.1 M NaOH. Obtained dispersions were incubated for 30 min at 30 °C in a water bath with continuous shaking. After incubation, the liquid fraction was carefully removed by centrifugation (5500× *g*, 20 min). The wet residue was weighted and the WAC was expressed as grams of water absorbed by the gram of sample (g/g). The supernatant was transferred to a glass tube and dried in an oven at 105 °C to constant weight (S). WS was calculated according to the following equation:

WS (%) = S × 100/W_0_ (1)

#### 2.9.2. Emulsifying Capacity and Emulsion Stability

The protein sample (0.5 g) was mixed with 10 mL of rapeseed oil in a graduated tube; further, the pH was adjusted to 4, 7, or 9 with 0.1 M HCl or 0.1 M NaOH. Protein dispersions were homogenized (IKA T5, Ultra-Turrax, Staufen, Germany) for 1 min and stored for 30 min at room temperature. After storage, the total volume (V_t_) and the volume of emulsified layer (V_em_) were fixed. Emulsifying capacity (EC) was defined as follows: EC (%) = V_em_/V_t_ × 100. Emulsion stability (ES) was measured by sample centrifugation at 3000× *g* for 5 min, following heating at 80 °C for 30 min, and was calculated according to the equation:

ES (%) = V_remaining em layer_/V_em_ × 100 (2)

#### 2.9.3. Foam-Forming Capacity and Foam Stability

Protein suspensions (10 mL, 4%, *w*/*v*) in distilled water after adjusting the pH to 4, 7, or 9 with 0.1 M HCl or 0.1 M NaOH were homogenized (IKA T5, Ultra-Turrax, Staufen, Germany) for 1 min in the graduated conical cylinders. The volume of starting liquid phase (V_L_) and the volume of foam formed immediately after mixing (V_F_) were fixed. The foamed samples were held for 30 min at room temperature to evaluate foam stability. The foam-forming capacity (FFC) and foam stability (FS) after 30 min storage were defined as follows:

FFC (%) = V_F_/V_L_ × 100, (3)

FS (%) = V_F30min/_V_F_ × 100. (4)

### 2.10. In Vitro Protein Digestibility

Digestibility in vitro was determined for the CMB prolamins according to the procedure described by Almeida et al. [19]. For analysis, the protein sample (0.5 g) was suspended in 20 mL of 0.1 M HCl, containing 1.5 mg/mL pepsin, and then incubated for 3 h at 37 °C in a water bath. After incubation, 10 mL of 0.5 M NaOH was added. Subsequently, 10 mL of 0.2 M phosphate buffer (pH 8.0) containing 10 mg of pancreatin was added. The protein solutions were incubated for 24 h at 37 °C. After the pancreatic hydrolysis, 1 mL of 10% TCA (trichloracetic acid) solution was added. Further, the protein solutions were centrifuged (8000× *g* for 20 min), and the nitrogen contents in the supernatant (N_S_) and the sample (N_t_) were measured. Protein digestibility (PD) was calculated:

PD (%) = (N_t_ − N_S_)/N_T_ × 100, (5)

where N_t_ and N_S_ represent the nitrogen content in the sample before and after digestion, respectively. All measurements were performed at least in triplicate.

### 2.11. Degree of Hydrolysis

The degree of hydrolysis (DH) of prolamins was measured according to Adler-Nissenn [20]. DH was expressed as a percentage ratio between the number of peptide bonds cleaved (h) and the total number of bonds available for proteolytic hydrolysis (h_total_):

DH = (B × N_b_/α × M_p_ × h_tot_) ×100%, (6)

where B: volume of NaOH solution (mL); α = 10pH-pK/1 + 10pH-pK; Mp: protein mass (g); N_b_: NaOH solution concentration (1 M); h_tot_ = 9.2 (for zein). All measurements were performed at least in triplicate.

### 2.12. Total Phenolic Content

Total phenolic content was determined in the extracts by the method of Singleton and Rossi [21] with slight modification. Lyophilized protein samples were dissolved in a 70% ethanol at a protein concentration of 1 mg/mL, and 0.5 mL of solution was added to 1.5 mL of freshly diluted (1:10) Folin–Ciocalteu reagent (2 N). The mixture was allowed to stand for 5 min, then 1.5 mL of sodium hydrocarbonate (75 g/L) was added. Afterwards, the mixture was incubated for 45 min in the dark and the absorbance read at 765 nm. The standard gallic acid solutions (0.01–1.0 mg/mL) were used for the construction of calibration curve. The results were expressed as mg gallic acid equivalent (GAE) per 1 g protein.

### 2.13. Determination of Antioxidant Activity

The scavenging activity of prolamins of untreated and pre-treated CMB samples was measured on 1,1-diphenyl-2-picrylhydrazyl (DPPH) free radicals according to the method of Tang and Zhuang [22]. Lyophilized protein samples were dissolved in a 70% ethanol at a protein concentration of 1 mg/mL, and 1 mL of sample solution was mixed with 1 mL of 0.1 mM DPPH solution in 95% ethanol. After shaking, the mixture was stored for 30 min at room temperature, and the absorbance of the sample solution was measured at 517 nm. Ethanol was used as a blank. The reference solution was prepared by mixing ethanol and DPPH solution. The antioxidant activity (AA) of prolamins was expressed as a percentage of DPPH radicals scavenged under the experimental conditions.

### 2.14. Statistical Analysis

All analyses except those of amino acids were performed at least in triplicate. The results are presented as mean values and standard deviations. The significant differences between means were assessed by analysis of variance (ANOVA) by Duncan test using the IBM SPSS Statistics 27.0 statistical package (SPSS Inc., Chicago, IL, USA). Data means were recognized as significantly different at *p* < 0.05.

## 3. Results and Discussion

### 3.1. Characterization of Stabilized CMB Material

The results of the comparative evaluation of chemical composition and physical and functional properties of CMB before and after extrusion are presented in Table 1 and Table 2, respectively. The fat, protein, crude fiber, and carbohydrate contents of CMB were 4.36, 12.14, 1.24, and 77.37 g/100 g dw, respectively. Results clearly showed that extrusion processing significantly (*p* < 0.05) affected the nutritional value of corn raw material: the extruded CMB contained 12.4% and 37% lower (*p* < 0.05) protein and fat contents, respectively (Table 1). On the other hand, the crude fiber, ash, and carbohydrate contents of the extruded CMB did not show a significant reduction compared to the raw material.

Our results regarding the effect of extrusion on the changes in cereal biopolymers during extrusion cooking are compatible with Hegazy et al. [11], who reported that the extrusion process caused a significant decrease in protein and fat contents of the corn-chickpea extrudates, while fiber, ash, and carbohydrates were not affected compared to the untreated material. A significant reduction of protein content can be due to the reaction occurring between amino groups of amino acids and carbonyl groups of reducing sugars in the presence of high temperature and pressure [23,24].

In the case of functional and safety properties of extruded CMB, the extrusion cooking (Table 2) caused a 5.3-log cycle decrease in the total microbial count, herewith significantly reducing the activity of endogenous xylanases and proteases (by 55 and 30.5%, respectively). The endogenous enzymes, such as xylanase and protease, play an important role in the digestibility of cereal nutrients. Xylanase disrupts the plant cell walls by hydrolyzing insoluble carbohydrates and simultaneously allows exogenous and endogenous enzymes to access proteins and other nutrients [25].

The obtained results indicated the increase in water-absorption capacity (WAC) as well as a degree of starch gelatinization of extruded CMB due to the significantly reduced (*p* < 0.05) particle size (mass density) and increased content of damaged starch as compared to CMB_UN_ (Table 2). According to the literature, extrusion cooking can cause the gelatinization of starch, protein denaturation, lipid separation [23], the complete or partial inactivation of microorganisms and enzymes, and an increase in soluble dietary fiber [24].

Extrusion, as a prevalent physical method causing starch pre-gelatinization, enhances its absorption and swelling power. During the extrusion process, starch is subjected to mechanical shearing at a high temperature and relatively low moisture. This process causes breakage of the covalent bonds between starch components, resulting in a strong structural destruction and partial depolymerization, which promotes the change of its functional properties [23].

The extent of structural and physicochemical changes of starch and proteinaceous components, such as endogenous enzymes and enzyme inhibitors, during extrusion primarily depends on the intensity of the extrusion process parameters. However, thermo-mechanical treatment even at 140 °C did not completely denature the proteins in wheat flour, which might be attributed to a lesser residence time of raw material within the extruder [23]. For example, extrusion temperature 143 °C led to partial inactivation (57%) of trypsin inhibitor in foods [26]. Thus, determining the optimal extrusion conditions for various parameters that will result in cereal products with higher nutritional value can be recommended.

### 3.2. The Influence of the Extrusion and Solid-State Fermentation on Protein Yield and the Retention of Amino Acids in CMB

#### 3.2.1. The Effect of Extrusion on the Protein Extraction Yields and the Amino Acid Profile

**Figure 1:**
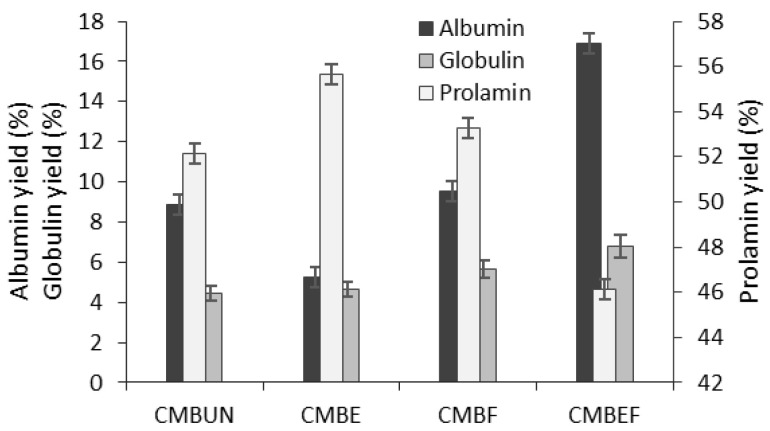
The yields of protein fractions isolated from the untreated (UN), extruded (E), and 48 h fermented (F) corn-milling by-products (CMB).

The yield of each protein fraction was expressed (Figure 1) as the percentage of the crude CMB protein content. After the isolation of three CMB protein fractions, the protein recovery was approximately 66.76% of crude protein content (12.14 g/100 g of CMB dw).

The Osborne solubility-based protein fractionation indicated that water-soluble albumins and salt-soluble globulins consist of about 14% of total proteins in CMB, while alcohol-soluble prolamin fraction showed the highest yield (52.17%) compared to albumins and globulins (8.87 and 4.47%, respectively) (Figure 1). Alkali-soluble glutelin fraction (not tested in this study) in corn consist by about 34% of crude proteins [27].

In our study, the most effective extrusion was noticed on the albumin and prolamin fractions as a result of heat and mechanical action. Total protein content (10.64 g/100 g dw) in extruded CMB was found significantly (*p* < 0.05) lower, and as was expected, prolamin yield (55.65%) was found significantly (*p* < 0.05) higher due to the reduced content of water-soluble albumins (5.23%), while the globulin proteins were found to be less sensitive to heat treatment during extrusion (yield 4.62%) (Figure 1).

As shown in Table 3, the most abundant amino acids in corn-milling by-products are GLU, LEU, PHE, VAL, ILE, THR, ASP, and PRO. Lack of some EAA, especially LYS, HIS, and TRP, and excess LEU can indicate the poor quality of proteins [27].

Extrusion cooking significantly (*p* < 0.05) reduced the NEAA and CEAA and also EAA contents in extruded samples (330.1 and 262.8 mg/g protein, respectively) compared to the untreated material (361.5 and 291.7 mg/g protein, respectively) (Table 3). Among EAA, which were affected, the most reduced were LYS (14.4%) and VAL (13.4%), following LEU, THR, and TRY (average 9.8%). Most contents of amino acids decreased significantly after extrusion due to the decomposition of amino acids into molecules of ammonia under the influence of high temperature and pressure.

In the case of EAA, the tendency is in accordance with the results reported by other authors for different cereals. Paes and Maga et al. [28] reported the reduction of the contents of ILE, LEU, LYS, THR, and VAL in whole-grain corn flour extruded in a single-screw extruder (130 °C; 80 rpm) compared to the raw material. For example, the losses of LYS may be due to the loss of albumins, which are rich in lysine, during the extrusion process [28]. According to Xiao et al. [29], extrusion of barley powder (140 °C, 40 kHz) decreased the LEU, GLU, and ARG contents. Perhaps, extrusion at low moisture and high temperature leads to starch degradation, thus providing contents of reducing sugars at the same time that it modifies protein structure and favors browning reactions. Since the ε-amino group of lysine has been referred to as a major reactant in the Maillard reaction, it might explain the extrusion effect on this particular amino acid.

#### 3.2.2. The Effect of SSF on the Improvement of Protein Yields and Amino Acid Profile

According to the results (Figure 1), SSF with *L. sakei* positively affected protein recovery: a slight increase in prolamin yield (2.1%) and significantly higher albumin and globulin contents (7.2 and 22.5%, respectively) were determined in fermented CMB compared to unfermented material (Figure 1). In extruded CMB after SSF (sample EF), the 2.2-fold higher average content of albumins and 46.9% higher level of globulins were determined, while the yield of prolamins (46.1%) was found significantly lower compared to the extruded CMB (55.65%) due to the proteolytic degradation occurred during the fermentation process (Figure 1).

This trend is in agreement with work of Cui et al. [30], demonstrating that fermentation of different maize cultivars in the presence of yeast caused a significant increase in protein content (43.5%), which was attributable to a decrease in carbon ratio in the total mass. The microorganisms utilize sugars as an energy source that causes the increased concentration of nitrogen in the fermentation medium and herewith the increase in the proportion of protein. Another possible explanation of this result might be an increase of microbial biomass during fermentation and thus an increase of total protein content. Overall, these trends were consistent with the results of protein analysis (Figure 1).

The amino acid profile is an important characteristic of evaluating the nutritional quality of protein in raw material. In our study, the quantities of most amino acids were increased in the untreated and extruded CMB after fermentation as a result of proteolysis, when peptides are broken down into amino acids by LAB-specific peptidases [30].

In the case of untreated CMB, the majority of EAA, such as LYS, TRP, HIS, VAL, ALA, and MET, contents increased after fermentation compared to unfermented CMB (Table 3). The greatest increase was fixed in LYS (34.0%), TRP (27.8%), HIS (18.9%), and MET (11.7%) contents. Moreover, the highest increase between NEAA and CEAA was determined for GLU (26.3%), TYR (17.8%), and CYS (10.2%).

For the extruded corn material, the amounts of all amino acids except ASP, CYS, SER, HIS, ARG, THR, and VAL increased after fermentation compared to unfermented extruded CMB. The contents of LYS (57%), TYR (33.7%), GLU (25.8%), and LEU (22.9%) increased the most after fermentation.

The results are consistent with the study of Thompson et al. [31], who found that the fermentation of beans and cauliflower increased the concentration of ALA, GLY, HIS, ILE, LEU, and VAL and also with Xiao et al. [29], reporting that lowered contents of GLU, GLY, ALA, and MET in barley powder after extrusion increased after fermentation due to the increased total protein content (Figure 1).

### 3.3. The Influence of Extrusion and SSF on CMB Albumin and Globulin Functional Properties

The results of the influence of extrusion on the albumin and globulin foam-formation capacity (FFC) and foam stability (FS) as well as the emulsifying capacity (EC) and emulsion stability (ES), water-absorption capacity (WAC), and solubility (WS) at different conditions (pH 4–9) are presented in Table 4.

#### 3.3.1. Solubility and Water-Absorption Capacity

WAC is a useful parameter to indicate the possibility of proteins being incorporated into food or feed formulations. The results indicated that the WAC values of CMB albumins and globulins depended on the pH of the medium and extrusion treatment (Table 4). The CMB albumins and globulins exhibited a relatively high WAC (2.12–2.64 g/g) comparable with the study of Gao et al. [32], demonstrating that extrusion improved the solubility of rice proteins and their water-holding capacity up to 37.7% after extrusion at 130 °C. Under the mechanical action, the protein molecular space structure increases due to the degradation of large molecules, making the water molecules easy to penetrate [32].

In our study, WAC for CMB albumins and globulins reached the maximum at pH 9, with values of 2.64 and 2.47 g/g, respectively. Singh et al. [33] reported the water-absorption of lyophilized corn proteins ranging from 2.8 to 3.13 g/g at pH 6.5. According to Pedroche et al. [34], the alkalinization of the protein solution has a positive effect on water absorption. In our study, the alkaline conditions resulted in a slightly lower WAC for globulins of extruded CMB without influencing albumin functionality (Table 4).

The albumin- and globulin-solubility profiles indicated most solubilization at alkali and neutral conditions than at acidic pH due to the low protein solubility close to their pI (4.1 and 4.3, respectively) [17]. There was not a significant difference at neutral and alkali conditions between the solubility of albumin and globulin fractions (64.8 and 63.5% (pH 7), and 73.7 and 72.3% (pH 9), respectively) as well as between solubility at pH 4 for both protein fractions (34.9 and 39.4%, respectively).

The obtained results suggest that CMB albumins and globulins have good solubility under basic conditions, which agrees with the results for the protein isolate obtained from jackfruit seeds [35]. Analogously, Lawal et al. [36] reported low solubility of African locust bean albumins (56.7%) at pH 5, while for globulins, the lowest solubility was observed at pH 4. According to the literature, at a pH > 6.5, the solubility of most plant proteins was reported to be higher than 70% [37,38].

The protein solubility at different pH values may serve as a useful indicator of the protein performance in the food systems in addition to the extent of protein denaturation affected by heat treatment. At the isoelectric point (pH 4–5), there is no net charge on the protein, resulting in no protein–protein interactions that are disadvantageous for the solubility.

The results indicated (Table 4) that extrusion improved water absorption of both protein fractions, increasing WAC values up to 2.72 and 2.61 g/g, respectively, at neutral (pH 7) conditions. Solubility of albumins and globulins was improved significantly (*p* < 0.05) in extruded samples at pH 7 and 9 (by 17%) but was only slightly improved at pH 4 for albumins (increase by 3%), and there was no significant effect found on globulins.

#### 3.3.2. Foaming Capacity and Foam Stability

As shown in Table 4, the foam-forming capacity (FFC) and foam stability (FS) of albumins and globulins significantly depended (*p* < 0.05) on the treatment and pH of the medium. The FFC was found to be high with values of 249–324% for albumins of untreated CMB; the lowest values were fixed at pH 4 and reached a maximum at pH 9. The FFC of the globulins was found at significantly (*p* < 0.05) lower levels (178–209%). Higher values of analyzed characteristics, appearing at alkali conditions, can be due to increased solubility of CMB proteins.

The maximum FS for corn albumins and globulins (69.6 and 64.8%, respectively) was observed at pH 7, whereas the lower FS was fixed at pH 9 (63.2 and 58.3%, respectively), and the lowest FS (~56%) occurred at pH 4. Ulloa and others [35] reported the maximum foaming capacity of 254% and FS of 164% for jackfruit seed protein at pH 10, whereas the minimum foaming capacity and FS occurred at pH 4; in addition, the FS was reduced at pH 8, compared to pH 6 and even pH 4. In contrast to these results, the foaming capacity and stability of lupin protein concentrate were found greatest at acidic pH values [39].

The foaming capacity of proteins is affected by changes in protein structure and solubility. According to Schwenzfeier and co-authors [40], the charges of soybean proteins changed with increasing pH, weakening the hydrophobic interactions and increasing protein flexibility. Thus, a faster protein diffusion to the surface of the air–water phase occurred, encapsulating air particles and increasing FFC. High foam stability of the protein in the isoelectric region is attributed to the formation of stable molecular layers in the air–water interface of the foams. At the pI of proteins, electrostatic forces reduced, lowering protein solubility and increasing surface tension; thus, the adsorption of proteins on the surface of foam bubbles become weak, resulting in a weaker foam formation and lower stability [32]. In the alkaline medium, the surface tension decreases, increasing foaming activity of proteins.

The results indicated that extrusion processing significantly (*p* < 0.05) reduced the FFC of albumin and globulin fractions to 215–256% and 141–179% and FS values to 53.8–65.1% and 52.1–60.3%, respectively (Table 4). According to Hojilla-Evangelista et al. [41], the foaming capacity and stability of dried corn germ albumin and globulin fractions at pH 7 and pH 10 were found to be of 124 and 130% and 84 and 92%, respectively. Maruatona and others [42], showing that after heat treatment (150 °C; 20 min), the foaming activity at pH 7 of bean flour decreased from 31.1 to 30.7%. The *Brassica carinata* protein isolate showed a foaming capacity of 280% at pH 10, and the foam stability after 20 min was 87% [34]. FFC is inferior to the extruded raw material proteins because the structure of the protein changes under the influence of heat and mechanical force, i.e., denaturation of proteins, causes the loss of their ability to form foam [43]. 

#### 3.3.3. Emulsifying Capacity and Emulsion Stability

The non-polar and polar amino acids cause hydrophobic and hydrophilic properties of proteins, leading to them acting as emulsifiers. The EC of CMB protein fractions depended on pH of the medium (Table 4), as it was observed to be the lowest under acidic conditions (pH 4) with values of 46.5 and 39.4% for albumins and globulins, respectively. It was consistent with the low solubility of proteins and high protein–protein interactions, which led to decreasing emulsion formation. At alkali conditions (pH 9), the EC of albumins and globulins significantly increased (*p* < 0.05) up to 59.6 and 49.5%, respectively, which were close to the Physic nut seed cake protein isolate [43]. Deb and others [44] reported the highest emulsifying capacity of 59.27% for the waste banana peel albumin fraction. According to Jayasena et al. [45], by increasing the pH values of lupine protein extracts from 4 to 8, the emulsifying capacity increased from 51 to 53.4%. Liu et al. [46] reported a pH-dependent increase in the emulsifying activity of soybean proteins, with the lowest value at pH 5.8 and the highest at pH 8. The high EC values of the CMB protein fractions accompanied its high solubility and thus rapid diffusion and adsorption at the interface. According to the literature data [47], emulsifying properties of Bambara bean protein isolate depended on the treatment temperature and pH.

As our study indicated, the EC of CMB albumin/globulin was the highest at pH 9, while emulsion stability was the highest at pH 4. According to Singh et al. [33], the emulsifying capacity and stability at pH 7 ranged from 42 to 53% and from 34 to 41% for corn albumins and globulins. Perilla seed proteins showed the lowest emulsifying activity at pH 4 but tended to increase at pH > 6 [48]. Emulsifying capacity of soluble proteins depends upon the hydrophilic–lipophilic balance, which is affected by pH. At acidic pH and close to the isoelectric point of proteins, the reduced net charges initiate the formation of stronger interfacial membranes; however, at pH 7, the presence of negative charges on the polypeptide chains leads to the formation of weak interfacial membranes, which would have the enhanced oil droplet coalescence and, consequently, reduced stability [42].

Extrusion of CMB significantly reduced the EC of soluble protein fractions (Table 4). The obtained trend is in line with Obatolu and co-authors [49], reporting that the formation of emulsions was highly weakened during thermomechanical processing. With increasing pH, the EC values were reduced by 12.5–25.7% for albumins and by 15.2–21.1% for globulins. The ES values of albumins and globulins after extrusion were the lowest at acidic conditions (39.3 and 32.7%). In the alkaline medium (pH 9), the ES of extruded albumin and globulin fractions were reduced by 23.4 and 14.4% compared to untreated samples (56.8 and 42.9%), respectively (Table 4).

#### 3.3.4. The Influence of SSF on the Changes of Extruded Albumins and Globulins Functional Properties

The results of the effect of SSF processing on the FFC and EC of albumins and globulins after extrusion are presented in Table 5. The analysis of the albumin and globulin functional properties was performed after 30 min retention at pH 9. As was expected, modification of extruded CMB by SSF positively affected the hydration properties and hydrophobicity of these protein fractions. Albumins and globulins in fermented extruded CMB showed WAC to be on average 27.9 and 13.8% higher, respectively, compared to proteins of unfermented extruded CMB (Table 4). The observed improvement of FFC and EC (on average 13.6 and 8.6%, respectively) for both protein fractions indicates the higher effect of fermentation on protein functional properties (Table 5).

Twenty-four-hour fermentation with *L. sakei* and *P. acidilactici* strains slightly improved ES (on average by 4.8%) and significantly increased FS (on average by 25.9%) of soluble proteins compared to unfermented samples. However, it should be noted that 48 h fermentation reduced FS of albumins and globulins by 14.5 and 22.8%, respectively, compared to 24 h fermentation. Further, the results showed that the hydration, emulsifying, and foaming capacity of albumins and globulins were slightly affected by the LAB strain used for fermentation, showing a stronger effect (*p* < 0.05) of *P. acidilactici* on globulins (Table 5). There was no significant difference (*p* ≥ 0.05) found between the different LAB used on albumin technological properties analyzed.

Worse functional properties in the case of extruded samples are a consequence of forming new intermolecular bonds, structures, and insoluble protein complexes. However, higher water-absorption ability should be connected with more protein released from protein complexes during fermentation [50]. Based on the literature, heat treatment by extrusion negatively affects the formation of protein emulsions, but it can be improved by applying fermentation. Obatolu et al. [49] demonstrated that lupin protein emulsifying activity after fermentation was increased by 23%, and similar results were obtained by Lampart-Szczapa [50], who showed higher lupin protein emulsion stability after solid-state fermentation with appropriate LABs.

Fermentation is a safe and green technology that uses the ability of LAB to produce organic acids and affects the structure of proteins. According to the literature, fermentation treatment resulted in an enhanced oil–water binding of sorghum protein by exposing hydrophobic groups inside the protein, thus improving its emulsifying properties [51]. The study by Tian et al. [52] showed that fermentation enhanced the surface electrostatic charge and solubility of egg yolk proteins, significantly improving its emulsifying activity.

### 3.4. The Effect of Extrusion and SSF on the Functional Properties and Bioactivity of CMB Prolamins

#### 3.4.1. Digestibility and Degree of Hydrolysis

**Figure 2:**
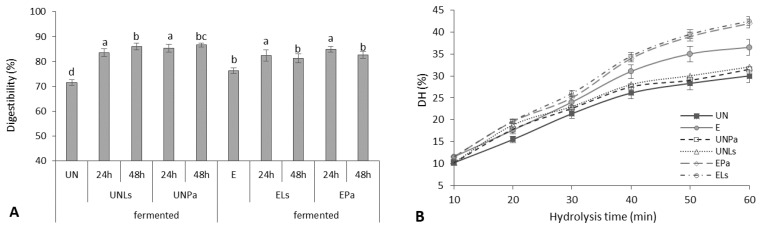
Digestibility (**A**) and degree of hydrolysis (DH) with trypsin (**B**) of prolamins isolated from untreated (UN) and extruded (E) CMB before and after fermentation (F) with *P. acidilactici* (Pa) or *L. sakei* (Ls). Different lowercase letters represent significant differences between data values (*p* < 0.05).

The digestibility of prolamins after SSF of untreated and extruded CMB with *L. sakei* and *P. acidilactici* varied from 83.56 to 86.69% and from 81.63 to 84.93%, respectively, compared to unfermented controls (71.61% and 76.32%, respectively) (Figure 2A), depending slightly on the LAB strain used for fermentation.

Extruded samples showed a significantly higher (by 20.4%) degree of hydrolysis (DH) of prolamins during 60 min hydrolysis compared to untreated CMB (Figure 2B). With increasing the time of hydrolysis with trypsin, the DH of extruded CMB prolamins was increased from 13.2% to 36.5% and for untreated CMB prolamins from 10.5 to 30%. The obtained trend was in line with Tang and Zhuang [22], reporting that the DH of corn zein fraction reached 24.5% using alkaline proteases and 15% using trypsin. According to the authors, the degree of hydrolysis tended to slightly decrease when increasing the hydrolysis time to 100 min [22].

The increase in the DH of extruded CMB proteins can be explained by the fact that structural changes in proteins occur due to the high temperature of 130–180 °C, pressure, and the screw of the extruder. During the extrusion of corn meal, the partially denatured proteins are stabilized by weak hydrogen bonding, electrostatic, and hydrophobic interactions [53], and such partial protein hydrolysis can improve protein digestibility [54]. As already mentioned, changes in the functional properties of protein fractions can be explained by the action of proteolytic enzymes and the formation of low-molecular-weight proteins and peptides.

The application of the SSF process additionally improved the DH of untreated CMB prolamins (5.8%) and significantly (*p* < 0.05) increased the DH of extruded CMB prolamins (on average by 13.6%). The results of the impact of fermentation on the digestibility of CMB proteins are close to the results of other authors. Krungleviciute et al. [55] reported an increase from 73% to 85% in the digestibility of lupine proteins after fermentation with *P. acidilactici* and *L. sakei* strains. Millet protein digestibility also was increased from 60.5 to 86.0% after 24 h fermentation [56]. It is believed that during fermentation, microorganisms produce different proteolytic enzymes [57] depending on the medium and process conditions, which can lead to an increase in protein digestibility.

#### 3.4.2. Antioxidant Activity of Prolamins

**Figure 3:**
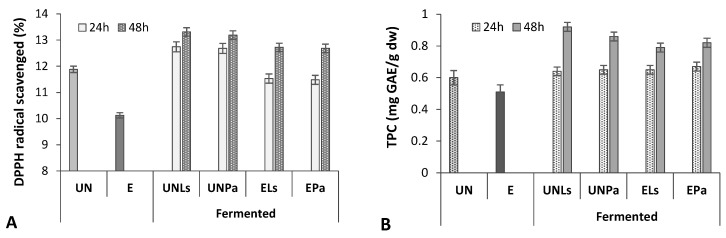
Antioxidant activity (DPPH) (**A**) and total phenolic content (TPC) (**B**) in prolamin fraction of untreated (UN) and extruded (E) CMB before and after fermentation with *L. sakei* (Ls) and *P. acidilactici* (Pa) strains.

The results of the influence of the extrusion and SSF on the antioxidant activity (AA) of prolamins (Figure 3A) showed that DPPH radical scavenging activity of extruded CMB prolamin fraction was considerably lower (14.7%) compared to untreated CMB prolamins. A slight increase in AA was found for the prolamins of untreated and extruded CMB after 24 h fermentation (6.5 and 8.3%, respectively). The 48 h SSF caused the significant increase (10.0% for CMB_UN_ and 20.2% for CMB_E_) in DPPH scavenging activity, indicating that the AA of CMB proteins partially lost during the extrusion cooking can be enhanced by the application of LAB fermentation process. Results also show that in the case of AA, there was not a significant difference (*p* ≥ 0.05) between LAB strains. In this case, more effective conditions to enhance the DPPH radical scavenging activity of extruded CMB, namely a 48 h fermentation with *L. sakei*, can be recommended (Figure 3A).

The AA values of hydrolyzed by SSF prolamins were comparable to the results reported in the literature. Yang et al. [10] reported a 31.60–56.98% scavenging ability of fermented by *Cordyceps militaris* 202 zein against DPPH. Tang and Zhuang [22] found zein alkaline protease hydrolysates exhibiting high antioxidant activity against DPPH (12.05%).

According to our study, LAB fermentation improved the AA of CMB prolamins mainly due to the release of bioactive compounds caused by bioacidification and LAB hydrolytic enzyme activity. This statement is consistent with the Cui et al. [30], demonstrating that SSF significantly increased total phenolic content (23.4%) of different maize cultivars. Moreover, free and bound phenolic compounds have been isolated from the wheat albumin, glutelin, prolamin, and globulin protein fractions [58].

Regression analysis of data obtained in our study confirmed that the increased antioxidant activity of prolamins correlated with the increased content of phenolics compounds (R^2^ = 0.6834) (Figure 3). Extrusion probably caused the breakdown of phenolics, while fermentation helped to degrade cell walls of dietary fiber as well as improve the extraction efficiency of phenolic compounds.

Stanisavljević et al. [59] reported that LAB fermentation with appropriate strains can exhibit strong proteolytic activity on pea proteins, producing high antioxidant active peptides. This indicates that protein hydrolysates contain components that are electron donors and could convert free radicals to stable form, depending on the molecular weight and hydrophobicity of the constituting peptides [60].

Overall, the study demonstrates that SSF with *L. sakei* and *P. acidilactici* is a potential method for the improvement of functional properties of CMB; moreover, such material can be used as a functional ingredient for food/feed applications with increased bioavailability and antioxidant potential of proteins.

## 4. Conclusions

Since corn is widely used for grits and snack production, large amounts of by-products rich in valuable proteins are inevitably generated in the production process; therefore, it is relevant to develop biotechnologies for the valorization of such cereal by-products to the food/feed ingredients. However, high-temperature extrusion changes significantly the feeding value of corn raw material. Thermo-mechanical treatment (extrusion) lowered fat and protein in CMB and also reduced protein fraction extraction yields, significantly reducing total amino acid content (9.2%). Among EAA, the most reduced were LYS and VAL, followed by LEU, THR, and TRY. However, it was demonstrated that SSF can partially improve the nutritional value of extruded CMB, positively affecting the recovery of protein fractions and increasing the EAA as well as total amino acids content on average by 12.5%, and improving protein functional properties. The study showed that 24 h fermentation significantly increased the hydration foaming and emulsifying capacity of corn albumins and globulins. The 48 h fermentation with tested LAB caused the significant increase in digestibility and DPPH scavenging activity of prolamins, indicating that the antioxidant activity partially lost during the extrusion cooking can be enhanced by the application of fermentation process.

The study demonstrates that the combination of extrusion-fermentation treatment can be confirmed as a prospective functionalization of CMB as feed/food stock capable of potentially reducing microbial contamination, enhancing its nutritional value, and improving technological properties of CMB albumins and globulins and digestibility and bioactivity of prolamins.

## Figures and Tables

**Table 1:** Chemical composition (g/100 g dw) of untreated and extruded corn-milling by-products (CMB).

CMB	Protein	Carbohydrates	Free Sugars	Crude Fiber	Fat	Ash
Untreated	12.14 ± 0.16 ^a^	77.37 ± 0.36 ^a^	1.13 ± 0.07 ^b^	1.24 ± 0.11 ^a^	4.36 ± 0.31 ^a^	4.89 ± 0.08 ^a^
Extruded	10.64 ± 0.11 ^b^	79.65 ± 0.64 ^a^	1.69 ± 0.06 ^a^	1.31 ± 0.08 ^a^	2.96 ± 0.17 ^b^	5.12 ± 0.06 ^a^

Results are the means ± standard deviation (n = 3). Data with different superscript letters within the column represent significant differences (*p* < 0.05).

**Table 2:** Physicochemical properties of untreated and extruded corn-milling by-products (CMB).

CMB Samples	TCM, log_10_ CFU/g	Mass Density, g/cm^3^	WAC, g/g	Damaged Starch, %	DG, %	Xylanase, XU/100 g dw	Protease, PU/100 g dw
Control	6.89 ± 0.21 ^a^	0.547 ^a^	2.24 ± 0.02 ^b^	33.6 ± 0.1 ^b^	48.8 ± 0.7 ^b^	77.8 ± 2.7 ^a^	33.4 ± 0.9 ^a^
Extruded	1.56 ± 0.19 ^b^	0.481 ^b^	3.82 ± 0.01 ^a^	44.2 ± 0.1 ^a^	59.9 ± 0.8 ^a^	34.7 ± 1.2 ^b^	23.2 ± 1.1 ^b^

Results are the means ± standard deviation (n = 3). Data with different superscript letters within the column represent significant differences (*p* < 0.05). TCM, total count of anaerobic microorganisms; DG, degree of gelatinization; WAC, water-absorption capacity.

**Table 3:** Amino acids (mg/g protein) in untreated (UN), extruded (E), and fermented (F) 48 h CMB.

Amino Acids	UN	E	F	EF
EAA				
Valine (VAL)	38.8 ^a^	33.6 ^b^	39.4 ^a^	30.4 ^c^
Isoleucine (ILE)	35.0 ^a^	32.2 ^b^	34.5 ^a^	32.8 ^b^
Leucine (LEU)	73.5 ^b^	66.2 ^c^	72.0 ^b^	81.4 ^a^
Tryptophan (TRP)	3.6 ^b^	3.2 ^c^	4.6 ^a^	4.3 ^a^
Lysine (LYS)	25.0 ^b^	21.4 ^c^	33.5 ^a^	33.6 ^a^
Methionine (MET)	16.3 ^c^	15.4 ^d^	18.2 ^b^	22.4 ^a^
Phenylalanine (PHE)	52.6 ^a^	48.2 ^b^	53.5 ^a^	46.7 ^b^
Threonine (THR)	33.2 ^a^	29.9 ^b^	33.4 ^a^	31.2 ^b^
Histidine (HIS)	13.7 ^b^	12.7 ^c^	16.3 ^a^	12.2 ^c^
Total EAA	291.7 ^b^	262.8 ^c^	305.4 ^a^	295.0 ^b^
NEAA and CEAA				
Alanine (ALA)	52.9 ^ab^	49.5 ^c^	54.4 ^a^	57.0 ^a^
Asparagine (ASP)	44.2 ^a^	40.4 ^ab^	42.5 ^a^	39.8 ^b^
Serine (SER)	24.3 ^a^	22.1 ^b^	25.2 ^a^	21.7 ^b^
Glutamine (GLU)	128.4 ^c^	116.5 ^d^	162.2 ^a^	146.6 ^b^
Cysteine (CYS)	7.8 ^b^	7.3 ^bc^	8.6 ^a^	7.5 ^b^
Proline (PRO)	33.7 ^a^	30.2 ^b^	35.2 ^a^	34.5 ^a^
Glycine (GLY)	22.3 ^b^	20.4 ^c^	24.7 ^a^	18.3 ^d^
Tyrosine (TYR)	28.6 ^b^	26.1 ^c^	33.7 ^a^	34.9 ^a^
Arginine (ARG)	19.3 ^a^	17.6 ^b^	20.9 ^a^	13.5 ^c^
Total NEAA and CEAA	361.5 ^bc^	330.1 ^d^	397.4 ^a^	373.8 ^b^
Total Amino Acids	653.2 ^b^	592.9 ^c^	712.8 ^a^	668.8 ^b^

Results are the means of two determinations. Data with different superscript letters within the row represent significant differences (*p* < 0.05). EAA, essential amino acids; NEAA, non-essential amino acids; CEAA, conditionally essential amino acids.

**Table 4:** Water-absorption capacity (g/g), water solubility, foaming and emulsifying capacity, and foam and emulsion stability (%) of untreated and extruded CBM albumins and globulins at different pH.

Samples	Albumins			Globulins		
	pH 4	pH 7	pH 9	pH 4	pH 7	pH 9
Untreated						
WAC	2.42 ± 0.12 ^bc^	2.52 ± 0.11 ^b^	2.64 ± 0.08 ^a^	2.12 ± 0.07 ^d^	2.27 ± 0.10 ^c^	2.47 ± 0.06 ^b^
WS	34.9 ± 0.1 ^c^	64.8 ± 0.2 ^b^	73.7 ± 0.3 ^a^	39.4 ± 0.1 d	63.5 ± 0.2 ^b^	72.3 ± 0.2 ^a^
FFC	249 ± 4 ^c^	266 ± 3 ^b^	324 ± 6 ^a^	178 ± 3 ^e^	186 ± 2 ^e^	209 ± 3 ^d^
FS	56.4 ± 0.5 ^d^	69.6 ± 0.7 ^a^	63.2 ± 0.4 ^b^	55.7 ± 0.6 ^d^	64.8 ± 0.3 ^b^	58.3 ± 0.2 ^c^
EC	46.5 ± 0.2 ^c^	52.2 ± 0.1 ^b^	59.6 ± 0.5 ^a^	39.4 ± 0.4 ^e^	43.8 ± 0.6 ^d^	49.5 ± 0.3 ^a^
ES	45.4 ± 0.8 ^c^	50.5 ± 0.7 ^b^	56.8 ± 0.2 ^a^	38.3 ± 0.1 ^de^	39.2 ± 0.4 ^d^	42.9 ± 0.5 ^d^
**Extruded**						
WAC	2.54 ± 0.13 ^b^	2.72 ± 0.14 ^a^	2.68 ± 0.11 ^a^	2.38 ± 0.08 ^c^	2.61 ± 0.12 ^b^	2.59 ± 0.10 ^b^
WS	37.6 ± 0.6 ^d^	78.2 ± 0.4 ^b^	88.6 ± 1.2 ^a^	38.7 ± 0.9 ^e^	76.7 ± 0.8 ^b^	87.4 ± 1.1 ^a^
FFC	215 ± 5 ^c^	238 ± 4 ^b^	256 ± 6 ^a^	141 ± 3 ^f^	152 ± 3 ^e^	179± 2 ^d^
FS	53.8 ± 0.2 ^c^	65.1 ± 0.6 ^a^	59.2 ± 0.8 ^b^	52.1 ± 0.7 ^d^	60.3 ± 0.4 ^b^	54.8 ± 0.3 ^c^
EC	40.7 ± 0.2 ^b^	42.8 ± 0.3 ^b^	44.3 ± 0.3 ^a^	33.4 ± 0.4 ^e^	35.5 ± 0.3 ^e^	38.9 ± 0.2 ^d^
ES	39.3 ± 0.1 ^b^	41.2 ± 0.2 ^b^	43. 6 ± 0.4 ^a^	32.7 ± 0.2 ^d^	34.2 ± 0.1 ^d^	36.7 ± 0.2 ^c^

Results are the means ± standard deviation (n = 3). Data with different superscript letters within the raw represent significant differences (*p* < 0.05). WAC, water-absorption capacity; WS, water solubility; FFC, foam-forming capacity; FS, foam stability; EC, emulsifying capacity; ES, emulsion stability.

**Table 5:** Water-absorption capacity (g/g), foaming and emulsifying capacity, and foam and emulsion stability (%) of extruded CBM albumins and globulins after fermentation with different LAB.

Samples	Albumins				Globulins			
	*L. sakei*		*P. acidilactici*		*L. sakei*		*P. acidilactici*	
	24 h	48 h	24 h	48 h	24 h	48 h	24 h	48 h
WAC	3.40 ± 0.12 ^a^	3.11 ± 0.09 ^b^	3.30 ± 0.10 ^a^	3.22 ± 0.07 ^ab^	2.89 ± 0.04 ^c^	2.92 ± 0.07 ^c^	2.96 ± 0.10 ^c^	3.02 ± 0.11 ^bc^
FFC	287 ± 2 ^a^	295 ± 2 ^a^	288 ± 2 ^a^	290± 2 ^a^	192 ± 1 ^d^	189 ± 1 ^d^	223 ± 2 ^b^	211 ± 2 ^c^
FS	78.8 ± 0.6 ^a^	68.7 ± 0.5 ^c^	82.4 ± 0.7 ^a^	69.2 ± 0.3 ^c^	72.9 ± 0.5 ^b^	56.8 ± 0.6 ^d^	73.5 ± 0.5 ^b^	56.1 ± 0.2 ^d^
EC	47.6 ± 0.3 ^a^	48.7 ± 0.5 ^a^	48.1 ± 0.6 ^a^	49.6 ± 0.4 ^a^	41.8 ± 0.2 ^b^	42.7 ± 0.5 ^b^	40.6 ± 0.7 ^b^	42.3 ± 0.6 ^b^
ES	46.5 ± 0.2 ^a^	47.0 ± 0.4 ^a^	46.0 ± 0.6 ^a^	47.5 ± 0.7 ^a^	37.6 ± 0.3 ^c^	39.8 ± 0.5 ^b^	38.9 ± 0.5 ^bc^	40.9 ± 0.7 ^b^

Results are the means ± standard deviation (n = 3). Data with different superscript letters within the raw represent significant differences (*p* < 0.05). WAC, water-absorption capacity; FFC, foam-forming capacity; FS, foam stability; EC, emulsifying capacity; ES, emulsion stability.

## Data Availability

Not applicable.
